# Retraction Note: SFRP2 augments WNT16B signaling to promote therapeutic resistance in the damaged tumor microenvironment

**DOI:** 10.1038/s41388-024-03233-8

**Published:** 2024-11-19

**Authors:** Y. Sun, D. Zhu, F. Chen, M. Qian, H. Wei, W. Chen, J. Xu

**Affiliations:** 1 Key Laboratory of Stem Cell Biology, Institute of Health Sciences, Shanghai Institutes for Biological Sciences (SIBS), Chinese Academy of Sciences (CAS) and Shanghai Jiaotong University School of Medicine (SJTUSM), Shanghai, China; 2 Collaborative Innovation Center of Systems Biomedicine, Shanghai Jiaotong University School of Medicine, Shanghai, China; 3 Department of Medicine and VAPSHCS, University of Washington, Seattle, WA USA; 4 Department of General Surgery, Zhongshan Hospital, Fudan University, Shanghai, China; 5 Department of Pharmacology, Changzheng Hospital, Second Military Medical University, Shanghai, China

Retraction to: *Oncogene* 10.1038/onc.2015.494, published online 11 January 2016

The Editor-in-Chief has retracted this article because the authors were not able to provide documentation of approval from an appropriate ethics committee. Additionally, concerns have also been raised about some of the data reported:

- In Figure 5c, the panels for Vehicle Control PSC27RAD and PSC27C appear to be duplicates.
- In Figure 6b, the panels for MIT-treated and Placebo-treated PC3/PSC27+IgG appear to overlap.

The Editor-in-Chief no longer has confidence in the reliability of this article’s finds and conclusions.

Y Sun has stated on behalf of all authors that they disagree with this retraction.

